# Supplementary material for: Machine Learning-Based Classification of Cervical Lymph Nodes in HNSCC: A Radiomics Approach with Feature Selection Optimization
Source: Cancers (Basel). 2025 Aug 20;17(16):2711. doi: 10.3390/cancers17162711 (PMC12384421; doi:10.3390/cancers17162711)
Supplement: Supplementary file 1 [file cancers-17-02711-s001.zip › cancers-3824706-supplementary.pdf]

Supplementary S1. Radiomics Features

Table S1 lists the radiomics feature classes and features extracted in this study (PyRadiomics).

Table S1. Radiomics feature classes and features included in the analysis. Abbreviations: GLCM, gray-level co-occurrence matrix; GLRLM, gray-level run-length matrix; GLSZM, gray-level size zone matrix; GLDM, gray-level dependence matrix; NGTDM, neighboring gray tone difference matrix.

Feature class	Features
First-order statistics	Energy; Total Energy; Entropy; Minimum; 10th Percentile; 90th Percentile; Maximum; Mean; Median; Interquartile Range; Range; Mean Absolute Deviation; Robust Mean Absolute Deviation; Root Mean Squared; Standard Deviation; Skewness; Kurtosis; Variance; Uniformity
Shape-based (3D)	Mesh Volume; Voxel Volume; Surface Area; Surface Area to Volume Ratio; Sphericity; Compactness1; Compactness2; Spherical Disproportion; Maximum 3D Diameter; Maximum 2D Diameter Slice; Maximum 2D Diameter Column; Maximum 2D Diameter Row; Major Axis Length; Minor Axis Length; Elongation; Flatness
Shape-based (2D)	Mesh Surface; Pixel Surface; Perimeter; Perimeter to Surface Ratio; Sphericity; Spherical Disproportion; Maximum 2D Diameter; Major Axis Length; Minor Axis Length; Elongation
GLCM	Autocorrelation; Joint Average; Cluster Prominence; Cluster Shade; Cluster Tendency; Contrast; Correlation; Difference Average; Difference Entropy; Difference Variance; Joint Energy; Joint Entropy; Informational Measure of Correlation 1; Informational Measure of Correlation 2; Inverse Difference Moment; Maximal Correlation Coefficient; Inverse Difference Moment Normalized; Inverse Difference; Inverse Difference Normalized; Inverse Variance; Maximum Probability; Sum Average; Sum Entropy; Sum Squares
GLSZM	Small Area Emphasis; Large Area Emphasis; Gray Level Non-Uniformity; Gray Level Non-Uniformity Normalized; Size Zone Non-Uniformity; Size Zone Non-Uniformity Normalized; Zone Percentage; Gray Level Variance; Zone Variance; Zone Entropy; Low Gray Level Zone Emphasis; High Gray Level Zone Emphasis; Small Area Low Gray Level Emphasis; Small Area High Gray Level Emphasis; Large Area Low Gray Level Emphasis; Large Area High Gray Level Emphasis
GLRLM	Short Run Emphasis; Long Run Emphasis; Gray Level Non-Uniformity; Gray Level Non Uniformity Normalized; Run Length Non-Uniformity; Run Length Non-Uniformity Normalized; Run Percentage; Gray Level Variance; Run Variance; Run Entropy; Low Gray Level Run Emphasis; High Gray Level Run Emphasis; Short Run Low Gray Level Emphasis; Short Run High Gray Level Emphasis; Long Run Low Gray Level Emphasis; Long Run High Gray Level Emphasis
GLDM	Small Dependence Emphasis; Large Dependence Emphasis; Gray Level Non-Uniformity; Dependence Non-Uniformity; Dependence Non-Uniformity Normalized; Gray Level Variance; Dependence Variance; Dependence Entropy; Low Gray Level Emphasis; High Gray Level Emphasis; Small Dependence Low Gray Level Emphasis; Small Dependence High Gray Level Emphasis; Large Dependence Low Gray Level Emphasis; Large Dependence High Gray Level Emphasis
NGTDM	Coarseness; Contrast; Busyness; Complexity; Strength

Supplementary S2. Normally Distributed Features

Seventy of the 120 features satisfied the Shapiro–Wilk normality test ($\alpha = 0.05$). Table S2 lists the normally distributed features (NDFs).

Table S2. Radiomics features following a normal distribution by the Shapiro–Wilk test ($p > 0.05$).

Feature class	Features (NDFs)
First-order statistics	Entropy; Minimum; 10th Percentile; 90th Percentile; Maximum; Mean; Median; Interquartile Range; Range; Mean Absolute Deviation; Robust Mean Absolute Deviation; Root Mean Squared; Skewness; Variance; Uniformity
Shape-based (3D)	Elongation; Flatness; Major Axis Length; Minor Axis Length; Maximum 3D Diameter; Maximum 2D Diameter Column; Maximum 2D Diameter Row; Surface Area to Volume Ratio; Sphericity
Shape-based (2D)	Elongation; Spherical Disproportion; Maximum 2D Diameter; Major Axis Length; Minor Axis Length
GLCM	Cluster Shade; Cluster Tendency; Correlation; Difference Average; Difference Entropy; Inverse Difference; Inverse Difference Moment; Inverse Difference Moment Normalized; Inverse Difference Normalized; Informational Measure of Correlation 1; Informational Measure of Correlation 2; Inverse Variance; Joint Average; Joint Energy; Joint Entropy; Maximal Correlation Coefficient; Max Probability; Sum Average; Sum Entropy; Sum Squares
GLSZM	Gray Level Non-Uniformity; Gray Level Variance; Low Gray Level Zone Emphasis; Size Zone Non-Uniformity Normalized; Small Area Low Gray Level Emphasis; Small Area Emphasis; Zone Entropy
GLRLM	Gray Level Variance; Long Run Emphasis; Low Gray Level Run Emphasis; Run Entropy; Run Percentage; Short Run Emphasis; Short Run Low Gray Level Emphasis; Run Length Non-Uniformity Normalized; Gray Level Non Uniformity Normalized
GLDM	Dependence Entropy; Dependence Variance; Gray Level Variance; Large Dependence Low Gray Level Emphasis; Low Gray Level Emphasis
